# Evidence that *Maackia amurensis* seed lectin (MASL) exerts pleiotropic actions on oral squamous cells to inhibit SARS-CoV-2 infection and COVID-19 disease progression

**DOI:** 10.21203/rs.3.rs-93851/v1

**Published:** 2020-10-23

**Authors:** Stephanie A. Sheehan, Kelly L. Hamilton, Edward P. Retzbach, Premalatha Balachandran, Harini Krishnan, Paola Leone, Gary S. Goldberg

**Affiliations:** 1. Department of Molecular Biology, and Graduate School of Biomedical Sciences, School of Osteopathic Medicine, Rowan University, Stratford, NJ 08084, USA.; 2. National Center for Natural Products Research, Research Institute of Pharmaceutical Sciences, School of Pharmacy, University of Mississippi, MS 38677, USA.; 3. Department of Physiology and Biophysics, School of Medicine, Stony Brook University Stony Brook, NY 11794-8661, USA; 4. Department of Cell Biology and Neuroscience, Cell and Gene Therapy Center, and Graduate School of Biomedical Sciences, School of Osteopathic Medicine, Rowan University, Stratford, NJ 08084, USA.

**Keywords:** COVID-19, SARS-CoV-2, oral squamous cells, Maackia amurensis, lectin, ACE2, infection, inflammation

## Abstract

COVID-19 was declared an international public health emergency in January, and a pandemic in March of 2020. There are over 23 million confirmed COVID-19 cases that have cause over 800 thousand deaths worldwide as of August 19th, 2020. COVID-19 is caused by the SARS-CoV-2 virus. SARS-CoV-2 presents a surface “spike” protein that binds to the ACE2 receptor to infect host cells. In addition to the respiratory tract, SARS-Cov-2 can also infect cells of the oral mucosa, which also express the ACE2 receptor. The spike and ACE2 proteins are highly glycosylated with sialic acid modifications that direct viral-host interactions and infection. *Maackia amurensis* seed lectin (MASL) has a strong affinity for sialic acid modified proteins and can be used as an antiviral agent. Here, we report that MASL targets the ACE2 receptor, decreases ACE2 expression and glycosylation, suppresses binding of the SARS-CoV-2 spike protein, and decreases expression of inflammatory mediators by oral epithelial cells that cause ARDS in COVID-19 patients. This work identifies MASL as an agent with potential to inhibit SARS-CoV-2 infection and COVID-19 related inflammatory syndromes.

## Background

SARS-CoV-2 has infected over 23 million people and caused over 800 thousand deaths around the world in just 9 months (as of August 2020)^1^. The SARS-CoV spike protein targets the angiotensin converting enzyme 2 (ACE2) receptor on host cells. This interaction is mediated by a receptor binding domain (RBD) in the S1 portion of the spike protein that recognizes the human ACE2 extracellular domain^2,3^. Transmembrane protease serine 2 (TMPRSS2) and furin cleave a polybasic sequence to unlink the S1 and S2 domains in the SARS spike protein to promote virial cell entry^3,4^.

Lung epithelium, primarily T2 but also T1 cells, are considered prime SARS-CoV-2 infection sites^5,6^. However other cells can be infected, including salivary gland and nasal epithelial cells^6–8^. ACE2 and furin protease are also highly expressed by human oral squamous epithelial cells of the mucosa and tongue where they can act as viral infection sites^9–11^. SARS-CoV-2 activates inflammatory pathways involving STAT3, IL6, and TNF that cause inflammation leading to pathologies including acute respiratory distress syndrome (ARDS)^12^.

The SARS-CoV-2 spike and host ACE2 proteins are both heavily glycosylated with sialic acids needed for viral infection. The SARS-CoV-2 spike protein has at least 22 N-linked glycosylation sequons per protomer, and about 15% of these glycans contain at least one sialic acid residue^3,13,14^. The human ACE2 receptor contains 7 N-linked and 3 O-linked glycans, and they all contain sialic acid residues^15^.

Lectins recognize specific glycosylation motifs, and can be used as antiviral agents^16^. In particular *Maackia amurensis* seed lectin (MASL) has a strong affinity for sialic acid modified proteins, and targets specific receptors to inhibit viral infection^16,17^, cancer progression^18,19^, and inflammation^20,21^. Indeed, the effect of MASL on oral squamous cell carcinoma is being investigated in Phase I human clinical trial^22^. However, effects of MASL on SARS-CoV-2 infection and inflammatory pathways have not been described. Here, we report that MASL targets the ACE2 receptor, inhibits SARS-CoV-2 spike binding, and decreases the expression of ACE2, furin, sialic acid glycosylases, and inflammatory cytokines in human OSCC cells. These data suggest that MASL offers an opportunity to target these cells topically and systemically by oral administration to help combat SARS-CoV-2 infection and disease progression.

## Methods

### Cell culture and imaging

HSC-2 cells were maintained in DMEM (Hyclone SH30021) supplemented with 25 mM HEPES (Hyclone SH30237) and FBS (Seradigm 1400–500) at 37°C in 5% CO2 and 100% humidity as described^18^. For biochemical analysis, cells at 80% confluence on 6 well tissue culture cluster plates (Falcon 353224) were exposed to MASL (Sentrimed) at concentrations and time periods indicated in text. Cells were then rinsed with PBS, transferred to microcentrifuge tubes with rubber policemen, pelleted at 14,000xg, aspirated, and frozen at −80°C. Cells cultured on 35mm poly-D-lysine-coated glass bottom culture dishes (MatTek P35GC-1.5–14-C) were used for live cell imaging. Cells were incubated for 1 hour in PBS with 0.4 mg/ml ACE2 antibody (Proteintech 66699-I-Ig) conjugated to Alexa Fluor 647 (Molecular Probes A30009) and 1.4 μM MASL (Sentrimed) conjugated to Alexa Fluor 595 (Molecular Probes A11005) to examine MASL and ACE2 localization. Cells were incubated for 1 hour in PBS with 2 μM SARS-CoV-2 spike protein (RayBiotech 230–30161) labeled with Alexa Fluor 555 (Molecular Probes A30007) with and without 1.4 μM MASL to examine the effect of MASL on spike binding. This glycosylated SARS-CoV-2 S1 spike protein (genbank QHD43416 Val16 - Gln690) is produced by HEK293 cells and migrates at ~120 kD and ~75 kD before and after glycosylase treatment. After incubation, cells were rinsed with PBS and examined by confocal microscopy on a Carl Zeiss Axio Observer Z1 equipped with a Plan-Apochromat 63X objective, apotome 2, filter sets to detect Alexa Fluor 555 and DIC with a Zeiss AxioCam Mrc camera Rev3 equipped with an INU series Tokai Hit Stage Top Incubator and Zen Pro 2.3 software as previously described^18,23^.

### RNA isolation and sequence analysis

Transcriptome analysis was performed as described^24^ and specified as follows. Total RNA was extracted from frozen cell pellets using Qiagen RNeasy Plus Universal mini kit according to manufacturer’s instructions (Qiagen, Hilden, Germany). RNA library preparations and sequencing reactions were conducted at GENEWIZ, LLC. (South Plainfield, NJ, USA). RNA samples were quantified using Qubit 2.0 Fluorometer (Life Technologies, Carlsbad, CA, USA) and RNA integrity was checked using Agilent TapeStation 4200 (Agilent Technologies, Palo Alto, CA, USA). RNA sequencing libraries were prepared using the NEBNext Ultra RNA Library Prep Kit for Illumina following manufacturer’s instructions (NEB, Ipswich, MA, USA). Briefly, mRNAs were first enriched with Oligo(dT) beads. Enriched mRNAs were fragmented for 15 minutes at 94 °C. First strand and second strand cDNAs were subsequently synthesized. cDNA fragments were end repaired and adenylated at 3’ends, and universal adapters were ligated to cDNA fragments, followed by index addition and library enrichment by limited-cycle PCR. The sequencing libraries were validated on the Agilent TapeStation (Agilent Technologies, Palo Alto, CA, USA), and quantified by using Qubit 2.0 Fluorometer (Invitrogen, Carlsbad, CA) as well as by quantitative PCR (KAPA Biosystems, Wilmington, MA, USA). Sequencing libraries were clustered on 1 lane of a flowcell. After clustering, the flowcell was loaded on the Illumina HiSeq instrument (4000 or equivalent) according to manufacturer’s instructions. The samples were sequenced using a 2×150bp Paired End (PE) configuration. Image analysis and base calling were conducted by the HiSeq Control Software (HCS). Raw sequence data generated from Illumina HiSeq was converted into fastq files and de-multiplexed using Illumina’s bcl2fastq 2.17 software. One mismatch was allowed for index sequence identification. Raw data were converted to transcripts per million (TPM) values used to calculate percent expression compared to untreated control cells. Data from this study can be accessed with BioSample accession SAMN14979424 (https://www.ncbi.nlm.nih.gov/biosample/14979424).

### Western blotting

Western blotting was performed as described previously^18,25^. Cells were lysed in lysis buffer (2% SDS, 10% glycerol, 10 mM EDTA, 50 nM DTT, 50 mM NaF, 0.2 mM Na_3_VO_4_, and 1 mM PMSF in 62.5 mM Tris pH 6.8), sonicated, and clarified by centrifugation. Protein (15 μg/lane) was resolved by SDS-PAGE, transferred to Immobilon-P membranes (Millipore IH1079562), and incubated with antisera specific for ACE2 (Proteintech 66699-I-Ig) and β-actin (Sigma A1978). Primary antibodies were recognized by secondary antibody conjugated to horseradish peroxidase (Invitrogen 31430) and detected using enhanced chemiluminescence (Thermo Scientific 32106). Signal was quantitated with ImageJ software. Membranes were stained with India ink to verify equal loading and transfer after blotting.

### Transcriptional reporter assays

Luciferase reporter constructs were transfected into Hela cells as previously described^26^. Pathways, targets, and inducers included Stat3 (TGCTTCCCGAATTCCCGAATTCCCGAATTCCCGAATTCCCGAATTCCCGAACGT) induced by IL6 (50ng/ml, R&D Systems 206-IL-010)^26^ and NFkB (GCTACAAGGGACTTTCCGCTGGGGACTTTCCAGG) induced by PMA^26,27^. Twenty four hours after transfection, cells were incubated with pathway inducers for 30 minutes, treated with MASL for 4–6 hours, lysed with One-Glo luciferase assay reagents (Promega), and luminescence was measured with a GloMax Multi+ detection system equipped with Instinct Software (Promega). Values were normalized to untreated HeLa control cells.

## Results and Discussion

SARS-CoV-2 spike proteins binds to ACE2 receptors on oral mouth and tongue epithelium to enable viral endocytosis, COVID-19 infection, and subsequent inflammation^28^. HSC-2 OSCC cells were used as a model system for this study. These cells were derived from the mouth floor of a 69 year old male and are HPV negative^18,29,30^. *Maackia amurensis* seed lectin (MASL) targets sialic acid modified receptors on these cells within 2 minutes of exposure^18,31^.

**Figure 1:**
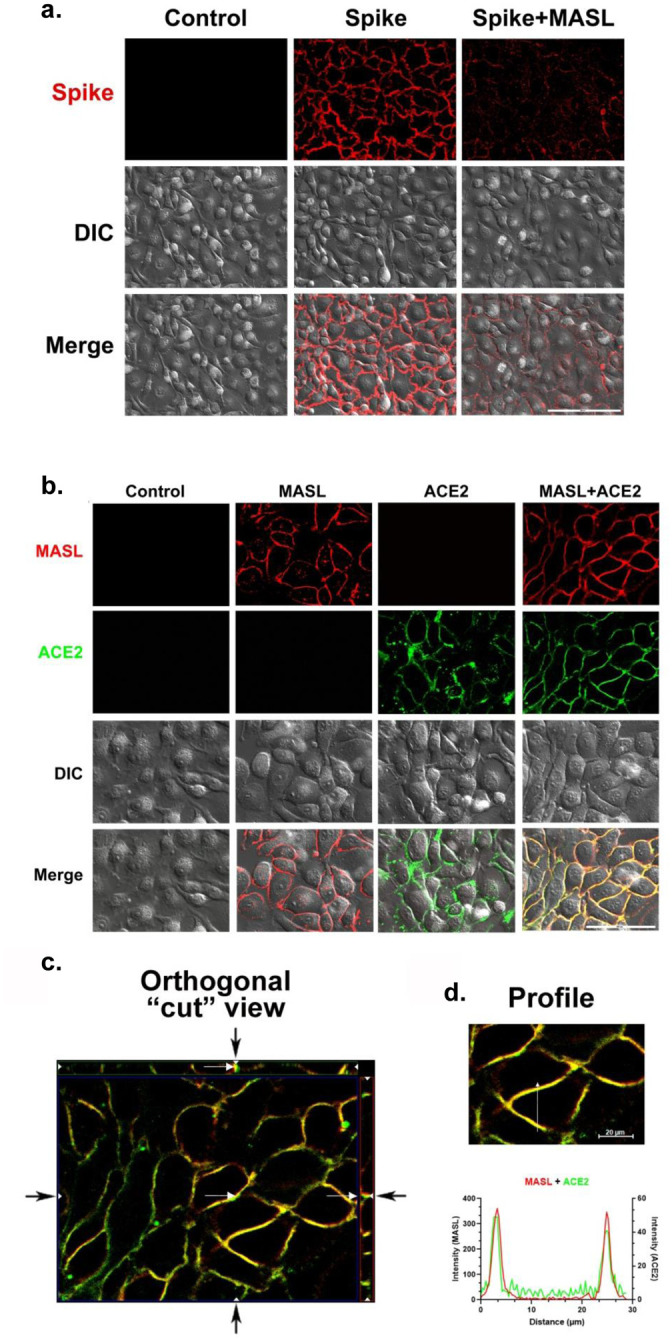
MASL colocalizes with ACE2 and inhibits SARS-CoV-2 spike protein binding to OSCC cells. **(a)** HSC-2 cells were incubated with 0.4 mg/ml Alexa 647 labeled ACE2 monoclonal antibody and 1.4 uM Alexa 595 labeled MASL and examined by live cell confocal microscopy. Fluorescent, DIC, and merged images are shown as indicated (bar=100 microns). **(b)** Orthogonal imaging of MASL and ACE2 colocalization in cut out view indicated by arrows (bar=20 microns). **(c)** Intensity plot profile over distance in one focal plane of an observed area as indicated. **(d)** Cells were incubated with 2 μM Alexa 555 labeled spike protein for 1 hour with and without 1.4 μM MASL. Fluorescent, DIC, and merged images are shown as indicated (bar = 200 microns).

Previous studies report that *Maackia amurensis* lectin binds to α2,3 and α2,6 O-linked sialic acid residues on host cell receptor glycoproteins to inhibit sapovirus infection^17^. The SARS-CoV-2 spike protein and human ACE2 receptor are both decorated with sialic acid residues needed for viral infection^3,13–15^. Therefore, we proposed that MASL can associate with the human ACE2 receptor and/or the SARS-CoV-2 spike protein to prevent infection. Results from live cell imaging experiments indicate that MASL colocalizes with the ACE2 receptor on HCS-2 cells as shown in Figure 1a–c. Accordingly, MASL effectively inhibited the ability of viral spike protein to target HSC-2 cells as shown in Figure 1d.

**Figure 2:**
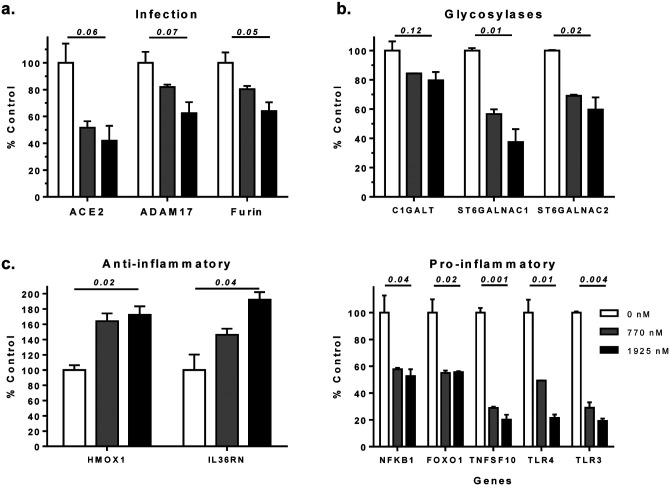
MASL affects expression of genes involved in SARS-CoV-2 infection and inflammation. HSC-2 cells were treated for 12 hours with 0, 770, or 1925 nM MASL and examined by RNA-Seq. Expression of gene transcripts were quantitated and shown as percent of untreated control cells (mean+SEM, n=2) with p values by ANOVA as indicated. **(a)** MASL inhibits ACE2, ADAM17, and furin mRNA levels. **(b)** MASL inhibits mRNA levels of glycosylases (C1galt, St6galnac1, and St6galnac2) needed for sialic acid modification of the ACE2 receptor. **(c)** MASL increases expression of anti-inflammatory transcripts (Hmox1 and Il36rn), and decreases expression of pro-inflammatory (Nfkb1, Foxo1, Tnfsf10, Tlr4, and Tlr3) mRNA transcripts.

**Figure 3:**
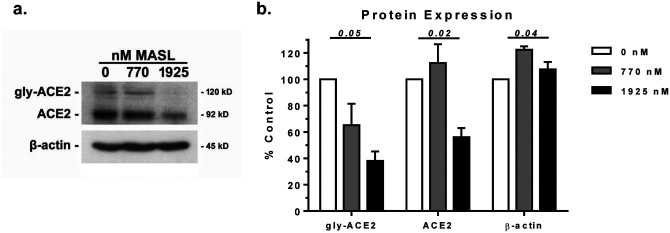
MASL inhibits ACE2 receptor expression and glycosylation. **(a)** HSC-2 cells were treated for 12 hours with 0, 770, or 1925 nM MASL and examined by Western blotting with apparent molecular weights shown as indicated. Primary and glycosylated ACE2 protein are evident at 92 and 120 kD, respectively **(b)** Protein expression was quantitated by image densitometry and shown as percent of untreated control cells (mean+SEM, n=3) with p values by ANOVA as indicated.

In addition to interfering with interactions between spike and ACE2 proteins, MASL appears to inhibit ACE2 expression and glycosylation. MASL decreases ACE2 mRNA levels in HSC-2 cells by nearly 50% and 60% at 770 nM and 1925 nM, respectively, as shown in Figure 2a. The human ACE2 receptor contains at least 10 moieties containing sialic acid (Neu5Ac or NANA) residues^15^. These modifications are catalyzed by the enzymes GalNAc-T, ST6GalNAc-1, and ST6GalNAc-2^32,33^. As shown in Figure 2b, MASL inhibits the expression of mRNA encoding these enzymes in a dose responsive manner. Taken together, these results suggest that MASL inhibits ACE2 expression and posttranslational sialic acid modification. These results are confirmed at the protein level by Western blotting. Treatment of cells with 1925 nM MASL for 12 hours inhibited ACE2 protein expression and glycosylation by over 50% as shown in Figure 3. In contrast, β-actin expression, which was used as a control, was either not affected or slightly increased (see Figure 3).

After viral recognition, furin protease cleaves the SARS-CoV-2 spike protein at the cell membrane to promote membrane fusion and viral endocytosis^3,4^. After furin cleavage, a disintegrin and metalloproteinase 17 (ADAM17) generates mature inflammatory ligands including IL6 in response to SARS-CoV-2 infection^34,35^. Interestingly, MASL decreased furin and ADAM17 mRNA levels in HSC-2 cells by nearly 20% and 40% at 770 nM and 1925 nM, respectively, as shown in Figure 2a.

**Figure 4:**
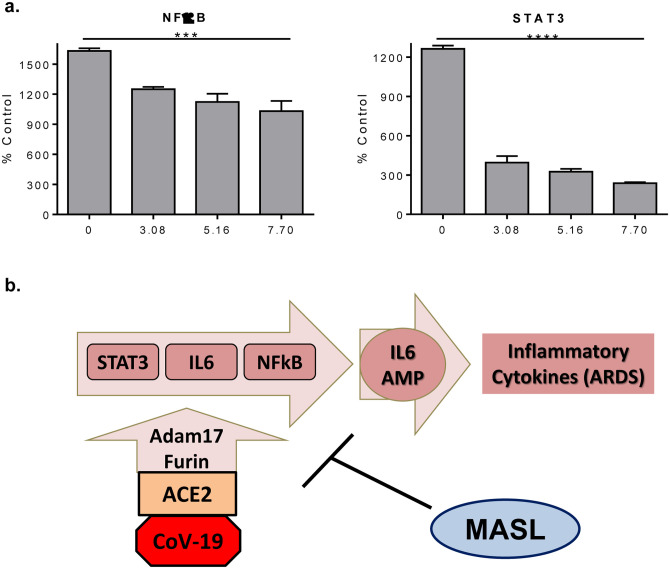
MASL affects NFκB and STAT3 transcriptional activation pathways. **(a)** HeLa cells transfected with Luciferase reporter constructs to detect NFκB and STAT3 activity were incubated with 0, 3.08, 5.16, or 7.70 μM MASL for 4–6 hours as indicated. Luminescence values were normalized to untreated nontransfected control cells and are shown as percent control (mean+SEM, n=2) with p values by ANOVA as indicated. **(b)** Diagram illustrating how MASL reduces ACE2, ADAM17, and furin expression, and decreases inflammatory signaling events that would otherwise lead to activation of the IL6 amplifier implicated in COVID-19 induced ARDS.

Once activated in response to infection, IL6 activates STAT3 in epithelial cells^34,36^. STAT3 signaling induces the expression of cytokines including more IL6 and NFκB^34,37^. IL6, STAT3, and NFκB cooperate to induce the IL6 amplifier (IL6-Amp) which hyper-activates NFκB to produce cytokines that cause multiple inflammatory responses^34,36^. This occurs in a variety of cells including chondrocytes^38^, intestinal^39^, lung^40,41^, and dermal epithelium^42^. NFκB can also induce IL6 production to induce vascular inflammation^43^. We utilized reporter transcriptional reporter assays to find that MASL inhibited STAT3 and NFκB signaling activity in a dose responsive manner as shown in Figure 4a.

NF-κB regulates cytokine expression and inflammatory immune response to infection^44^. Accordingly, NF-kB signaling has been implicated in the control of ACE2 expression and COVID-19 inflammatory pathologies^34,45,46^. If left unchecked, these infections induce FOXO1 expression in epithelial cells including oral mucosa, which induces the expression of toll-like receptors (TLRs)^47^. TLR signaling induces interleukin-36 (IL36) production, which induces IL6 expression^48^. IL6 then goes on to produce inflammatory cytokines in response to infections including tuberculosis in lung epithelial cells^40,41^. Heme oxygenase 1 (HMOX1) induces IL36RN expression, which acts as an IL36 antagonist to inhibit inflammation^49,50^. As shown in Figure 2c, MASL increased both HMOX1 and IL36RN mRNA expression in a dose responsive manner. These data suggest that MASL utilizes HMOX1 to induce IL36RN expression. Along with increasing the expression of anti-inflammatory mediators, MASL also decreased the expression of mRNA encoding inflammatory transcription factors NFκB and FOXO1, as well as the inflammatory cytokine TNFSF10, and toll-like receptors TLR3 and TLR4 in a dose responsive manner as shown in Figure 2c.

SARS-CoV-2 kills about 2% of infected individuals. This mortality rate is over 10 times higher than that of the seasonal influenza virus^51^. Severe acute respiratory distress syndrome (ARDS) is a major COVID-19 morbidity^52^. COVID-19 instigates chronic inflammation resulting in a “cytokine storm” that causes most ARDS mediated deaths^53–55^. COVID-19 also causes multisystem inflammatory syndrome (MIS) in children and adolescents. This hyper-inflammation leads to multiple organ failure and shock^56^. Treatments for these inflammatory syndromes include parenteral immunoglobulin and steroids with limited efficacy^57^. The IL-6 antibody blocker tocilizumab was found an effective treatment for CAR-T cell induced cytokine storm, and has been adopted as a treatment for COVID-19 inflammation^58–60^.

Unlike antibodies, lectins can be taken orally to treat diseases^61–63^ including cancer^61^ and viral infections^16^. MASL targets sialic acid modified receptors to inhibit cancer progression and inflammation^18,19,21^. Results from this study indicate that MASL inhibits ACE2 expression, SARS-CoV-2 spike binding, and major components of the IL6 amplifier including STAT3, IL6, and NFκB as illustrated in Figure 4b. In addition to viral lung inflammation, IL6 signaling also triggers contact dermatitis and psoriasis in keratinocytes^42^, as well as arthritic inflammation in chondrocytes^38^. COVID-19 inflammation shares inflammatory mechanisms with arthritis^12^. Indeed, COVID-19 infection causes arthralgia and myalgia in 15% and 44% of patients, respectively^64,65^. It should therefore be noted that MASL has been found to attenuate inflammatory NFκB signaling and inflammation in chondrocyte cell culture, and can be administered orally to alleviate arthritis progression in mice^21^. In addition, MASL has also been reported to suppress interleukin induced psoriatic inflammation in reconstituted epidermis^66^. Taken together, data suggest that MASL has the potential to be used alone or in combination with other antiviral and anti-inflammatory agents for COVID-19 treatment.
